# Models for Understanding Resistance to Chemotherapy in Liver Cancer

**DOI:** 10.3390/cancers11111677

**Published:** 2019-10-29

**Authors:** Jose J. G. Marin, Elisa Herraez, Elisa Lozano, Rocio I. R. Macias, Oscar Briz

**Affiliations:** 1 Experimental Hepatology and Drug Targeting (HEVEFARM), University of Salamanca, IBSAL, 37007 Salamanca, Spain; elisah@usal.es (E.H.); elisa_biologia@usal.es (E.L.); rociorm@usal.es (R.I.R.M.); obriz@usal.es (O.B.); 2 Center for the Study of Liver and Gastrointestinal Diseases (CIBERehd), Carlos III National Institute of Health, 28029 Madrid, Spain

**Keywords:** chemoresistance, cholangiocarcinoma, hepatoblastoma, hepatocellular carcinoma, multidrug resistance, resistome

## Abstract

The lack of response to pharmacological treatment constitutes a substantial limitation in the handling of patients with primary liver cancers (PLCs). The existence of active mechanisms of chemoresistance (MOCs) in hepatocellular carcinoma, cholangiocarcinoma, and hepatoblastoma hampers the usefulness of chemotherapy. A better understanding of MOCs is needed to develop strategies able to overcome drug refractoriness in PLCs. With this aim, several experimental models are commonly used. These include in vitro cell-free assays using subcellular systems; studies with primary cell cultures; cancer cell lines or heterologous expression systems; multicellular models, such as spheroids and organoids; and a variety of in vivo models in rodents, such as subcutaneous and orthotopic tumor xenografts or chemically or genetically induced liver carcinogenesis. Novel methods to perform programmed genomic edition and more efficient techniques to isolate circulating microvesicles offer new opportunities for establishing useful experimental tools for understanding the resistance to chemotherapy in PLCs. In the present review, using three criteria for information organization: (1) level of research; (2) type of MOC; and (3) type of PLC, we have summarized the advantages and limitations of the armamentarium available in the field of pharmacological investigation of PLC chemoresistance.

## 1. Introduction

**Figure 1:**
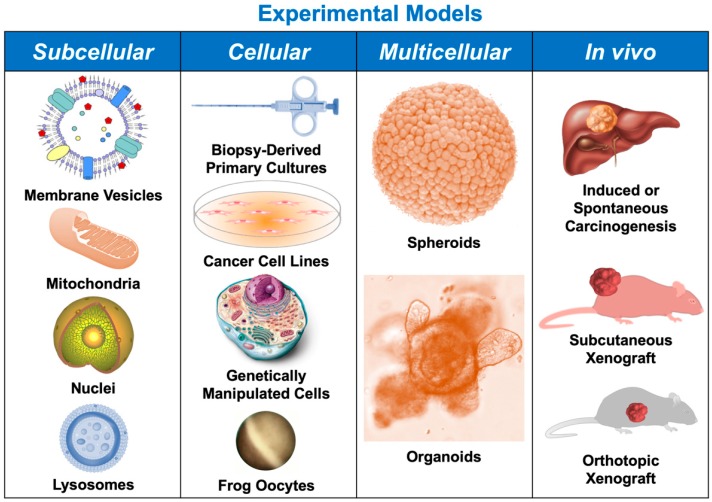
Schematic representation of experimental levels and models used in the investigation of mechanisms of resistance to chemotherapy in liver cancer.

Hepatocellular carcinoma (HCC) is the most frequent primary liver cancer (PLC), representing approximately 90% of PLCs in adults, whereas the remaining cases are mainly intrahepatic, ductular, and perihilar cholangiocarcinoma (CCA); hepatoblastoma (HB) being the most common type of PLC in pediatric patients. A critical limitation in the management of advanced PLCs is their poor response to pharmacological treatment, which is less inefficient in the case of HB. The mechanisms of chemoresistance (MOCs) accounting for this marked multidrug resistance (MDR) phenotype have been classified into seven groups from MOC-1 to MOC-7 [1]. Thus, even before starting the treatment, several of the approximately one hundred genes involved in these MOCs are already expressed. This results in a group of proteins that defines the intrinsic ‘resistome’, which accounts for the initial defense of the tumor against chemotherapeutic agents. Interestingly, some of these genes are shared among HCC, CCA, and HB [2]. Moreover, the resistome profile changes in response to the pharmacological challenge as a Darwinian adaptation of cancer cells, in which cancer stem cells (CSCs) play a crucial role, leading to induced chemoresistance and tumor growth relapse after the initial apparent response. To investigate the role of individual genes and the synergic interactions among MOCs, several experimental models (Figure 1), reviewed below, have been used.

## 2. Subcellular Models

### 2.1. Plasma Membrane

Membrane preparations, mainly plasma membrane vesicles (PMVs), are a simple system used to obtain information about the structure and function of carriers with the advantage of allowing investigation of the transport process without the influence of other cellular activities, such as drug metabolism (MOC-2). Thus, PMV transport assay is used to directly measure the translocation of substrates by uptake carriers (MOC-1a) by right-side-out PMVs and ATP-binding cassette (ABC) transporters (MOC-1b) by inside-out PMVs, in which the vesicles have the binding sites for both ATP and the substrate of the transporter in contact with the incubation buffer [3]. The substrates, usually radiolabeled derivatives, are taken up by PMVs in an ATP-dependent manner. As a limitation, only the activity of transporters present in the inside-out, but not in right-side-out, sealed vesicles can be measured. Finally, the vesicles are separated from the incubation medium by filtration and the amount of compound trapped within the vesicles can be quantified, usually by liquid scintillation. This assay cannot be used to test the transport of compounds that are highly permeable to the plasma membrane because the high rate of passive diffusion reduces the amount of compound retained by PMVs [4].

Several methods have been developed to obtain PMVs from cells and tissues [5]. Crude membrane preparations usually have approximately an equal proportion of vesicles with each orientation, mainly derived from the plasma membrane, although they also contain contamination by intracellular membranes [6]. This experimental model has contributed to advance in the identification of antitumor drugs that are substrates of the main ABC pumps involved in MOC-1b of HB and HCC, such as multidrug resistance protein 1/P-glycoprotein (MDR1/P-gp) (*ABCB1*) [7] and MRP2 (*ABCC2*) [8], and CCA, such as MRP3 (*ABCC3*) [9].

Using PMVs, indirect transport assays can also be carried out to determine the ability of the tested compounds to modulate the transport of well-known substrates. For example, PMVs have been used in the screening of MRP2 inhibitors through their ability to reduce the transport of carboxy-fluorescein [10]. However, this method does not provide information on whether the tested compound is actually transported or acts only as an inhibitor.

The sources to obtain PMVs include: (i) baculoviral/insect cell expression systems, whose main advantages are the high degree of protein expression and the existence of post-translational modifications similar to those occurring in mammalian cells. However, the lower cholesterol content in the plasma membrane of insect cells and the different glycosylation pattern of plasma membrane glycoproteins could have functional consequences on the heterologously expressed transporter. Thus, cholesterol-rich vesicles of Sf9 cells derived from *Spodoptera frugiperda* have been used to study drug efflux through the pump BCRP (*ABCG2*), which is more abundantly located in cholesterol-rich raft domains of the plasma membrane [11]; (ii) mammalian cancer cell lines (CCLs) stably expressing the desired transporter, although these rarely achieve the high level of protein expression that is required to carry out measurable transport by PMVs [6]; (iii) purified ABC transporters packaged in artificial PMVs such as liposomes, which are less frequently used due to the technical difficulties encountered in the generation of this model [12,13].

The ATPase assay that indirectly measures the activity of ATP-dependent transporters can be used with crude membrane preparations containing both inside-out sealed vesicles and lamellae or leaky vesicles [14] to determine whether the tested compound acts as a substrate and/or inhibitor [6]. In the presence of these transporters, the rate of ATP cleavage is proportional to the transport activity [15,16,17,18]. Transported substrates typically increase ATPase activity, while inhibitors decrease the initial activity and/or that measured in the presence of stimulating agents. This test has been used in the screening of potential inhibitors for MDR1 and BCRP [19]. For example, the ability of tyrosine kinase inhibitors such as cabozantinib [20], tariquidar derivatives [21], biscoclaurin alkaloids [22], and lanostane-type triterpenoids [23] to inhibit these pumps in PMVs obtained from HCC and HB cell lines with MDR phenotype has been demonstrated using this approach.

A drawback of this method is the inaccuracy of the results when studying compounds that are translocated very slowly by the carrier, as is the case of cyclosporine A transport by MDR1 [24], which does not generate a detectable accumulation of inorganic phosphate from ATP hydrolysis. This limitation can be overcome using the nucleotide trapping assay in inside-out and leaky vesicles. This test is based on the fact that during the function of ATP-dependent pumps, a transition state complex containing ADP occluded at the nucleotide-binding site is formed and can be stabilized with some compounds such as vanadate, fluoro-aluminate, and beryllium fluoride [25]. Therefore, the nucleotide trapping assay measures the amount of nucleotide linked to the binding site by trapping agents as a reflection of the transport rate, which increases in the presence of substrates and is reduced by some inhibitors [26].

Interestingly, hepatic cells—including hepatocytes, cholangiocytes, and CCLs—can spontaneously release extracellular vesicles [27], which are heterogeneous particles that include exosomes or small membrane vesicles (30–150 nm diameter), originated from the endosomal compartment, and larger extracellular microvesicles (150–1000 nm diameter) directly derived from plasma membrane [28]. Extracellular vesicles appear to be involved in drug detoxification (MOC-2) and the regulation of the tumor microenvironment (MOC-6). Moreover, the uptake of extracellular vesicles released by cancer cells with a marked MDR phenotype can confer resistance to other tumor cells [28].

### 2.2. Mitochondria

The isolation of mitochondria [29] has permitted the study of physiological functions—such as energy metabolism, identification of the mitochondrial genome, mechanisms of apoptosis, and mitochondrial transport—but has also served as a model for the development of pro-apoptotic antitumor drugs targeted to this organelle and the study of cancer chemoresistance [30]. Regarding MOC-1b, several ABC proteins, such as MDR1 [31] and BCRP [32], have been detected in mitochondria isolated from HCC derived cells (PLC/PRF/5) with MDR phenotype. These proteins were functionally active and able to transport antitumor drugs such as doxorubicin (MDR1 substrate) and mitoxantrone (BCRP substrate), from the mitochondria to the cytosol, protecting mitochondrial DNA of HCC cells from the damage caused by genotoxic agents. The function of TOP1MT, the only exclusively mitochondrial topoisomerase, has also been studied with mitochondrial preparations isolated from HCC cell lines. TOP1MT is involved in relaxing mitochondrial DNA during replication and transcription. This protein is a target of topoisomerase inhibitors such as camptothecins and anthracyclines (MOC-3). In HCC, the level of TOP1MT expression has been proposed as a prognostic marker for the response to chemotherapy [33]. In HCC-derived CCLs with MDR phenotype, the translocation from the nucleus to the mitochondria of telomerase reverse transcriptase (TERT) is increased, which could have an antiapoptotic role (MOC-5), increasing chemoresistance [34]. Regarding changes in the tumor microenvironment (MOC-6), an increase in the expression of inducible nitric oxide synthase has also been found in mitochondria isolated from HCC cells [35], which is known to increase the angiogenic activity in tumors. This experimental model has also been used for the evaluation of mitochondria-targeted drugs aimed to overcome chemoresistance, such as palladium coordination complexes that bind to thiol groups of mitochondrial membrane proteins and induce apoptosis [36].

### 2.3. Nuclei

Isolated hepatocyte nuclei have been found to keep intact the transport processes that occur across the nuclear membrane [37]. The presence of MRP2 at the external layer of the nuclear envelope has been reported [38]. In combination with the activity of phase II enzymes, this export pump could be involved in the nuclear barrier that protects genomic DNA from genotoxic compounds [38]. Preparations of nuclei suspensions isolated from HCC and HB cells have also been used to study alterations in the interaction between drugs and their targets (MOC-3) [39], as well as survival and apoptosis pathways (MOC-5) [40].

### 2.4. Lysosomes

Owing to their heterogeneity, low number, and marked fragility, the purification of lysosomes is a difficult task [41]. Lysosomes are capable of storing hydrophobic drugs with weak base characteristics by a mechanism that seems to be mediated by ABC proteins of the A subfamily [42], which produces a decrease in the concentration of the active antitumor drug in the cytoplasm [43]. This process and its role in resistance to tyrosine kinase inhibitors, such as imatinib and sunitinib, have been studied in leukemia and in immortalized hepatocytes (Fa2N-4) [44].

## 3. Cellular Models

Numerous cellular models have been developed to investigate chemoresistance and evaluate strategies for overcoming this problem. Immortalized CCLs are one of the most popular of these in vitro models. They have several advantages, which include the retention of the hallmarks of primary cancer cells, cellular homogeneity, accessibility to genetic manipulation, and reproducibility of results. At present, there are available over 30 CCLs derived from HCC [45], 15 from HB [46], and more than 50 from CCA [47], with different genetic alterations and degrees of sensitivity to anticancer drugs. A useful variant to investigate MOC activation is the use of drug-resistant CCLs, developed through repeated and increasing exposure to drug-induced selective pressure. Other cellular models of acquired chemoresistance have been developed by genetic manipulation of wild-type CCLs, such as gene amplification, deletion, or point mutations.

The expression profiles of genes involved in chemoresistance in HCC, CCA, and HB cell lines have revealed cell-specific patterns together with characteristic changes in response to the chemotherapeutic challenge [2]. A shared feature of HCC, CCA, and HB cells is the low expression of transporters of the solute carrier family [2,48,49,50], which results in a reduction of the intracellular levels of the active agents. Using CCLs, impaired expression and/or function of OCT1 (*SLC22A1*), involved in the uptake of cationic drugs, such as sorafenib, by HCC and CCA cells has been found [51]. Heterogeneous functional expression of ABC export pumps has been observed in several CCLs. Thus, high levels of MRP1-4 were found in HB cells [50,52], which contribute to its MDR phenotype. In HepG2 cells, with induced MDR1 overexpression, the relevance of this export pump in the resistance to doxorubicin has been confirmed [53].

Using CCLs, BCRP has been reported as one of the determinants of HCC chemoresistance to irinotecan [54]. Moreover, when resistance to cisplatin was induced in these cells, an increase in the expression of MRP4 has been observed [2,55]. In CCA cells, MDR1 is highly expressed [56] and MRP3 levels have been correlated with resistance to etoposide, doxorubicin, and pirarubicin [56].

Concerning changes in drug metabolism by liver CCLs, which include either lower ability to activate prodrugs or enhanced detoxifying capability (MOC-2), the overexpression of UDP-glucuronosyltransferase1A1 (UGT1A1), responsible for SN-38 detoxification, and hence resulting in lower sensitivity to this drug, has been found both in HCC [54] and HB [57] cells. NAD(P)H-quinone oxidoreductase 1 (NQO1)—a critical phase I detoxifying enzyme involved in the chemoresistance to 5-fluorouracil (5-FU), doxorubicin, or gemcitabine—is also overexpressed in CCA cells [58]. A marked change observed in PLC cells as compared to healthy liver cells is the upregulation of the placental isoform of glutathione-S-transferase (GSTP1) [2]. This enzyme can neutralize many antitumor drugs by conjugation with glutathione. GSTP1 silencing or specific pharmacological inhibition results in reversal of the resistance to doxorubicin, cisplatin, and several alkylating agents [59].

Changes in the molecular targets of anticancer drugs can also result in an impaired response of HCC, CCA, and HB cells to chemotherapy (MOC-4). Thus, mutations in the α-tubulin gene have been associated with lower sensitivity to *Vinca* alkaloids in HCC cells [60]. Moreover, TOP2A has been found upregulated in doxorubicin-resistant HCC cells [61].

Genes involved in the balance between pro- and anti-apoptotic factors are altered in many liver CCLs, which dramatically affects their response to pharmacological treatments (MOC-5). For instance, using HCC cells, p53 harboring mutation p.R248Q was reported to be accompanied by resistance to doxorubicin and paclitaxel [62]; in 5-FU-resistant HCC cells, which were also cross-resistant to vincristine, doxorubicin, and paclitaxel, transfection with wild-type p53 restored apoptosis-activation in response to these drugs [63]. Moreover, long-term exposure to irinotecan reduces the expression of p53, enhancing the tolerance of Huh-7 cells to this drug [64]. Another example concerns the inhibitor of apoptosis protein survivin (*BIRC5*); its upregulation, together with the downregulation of the pro-apoptotic factor TP73, was associated with 5-FU resistance in CCA cells [65]. Bcl-2 and Bcl-x(L) are also essential players in MOC-5 of HCC and CCA cells. Thus, in SNU-398 cells derived from HCC, Bcl-x(L) expression was induced during treatment with paclitaxel [66], whereas in CCA cells, Bcl2 upregulation, together with Bax downregulation, has been associated with resistance to 5-FU, cisplatin [67], and gemcitabine [68]. Another anti-apoptotic member of the Bcl-2 family, myeloid cell leukemia-1 (Mcl-1) has been found upregulated in several HCC cells, such as Huh-7, which significantly limits the response to 5-FU and valproic acid [69].

Chemoresistance due to phenotype transition (MOC-7) has also been identified in HCC, CCA, and HB cells. Poor differentiated CCLs, such as HLE and HLF, lose E-cadherin, express mesenchymal markers such as N-cadherin, and are more invasive and resistant to cisplatin, doxorubicin, and sorafenib than more differentiated CCLs, such as Hep3B, HepG2, and Huh-7 [70].

The use of CCLs also has limitations, such as genomic instability and the absence in the culture of the specific tumor microenvironment. An alternative to overcome these drawbacks is the use of primary cultures of cells directly isolated from PLCs, often at the time of diagnosis, whose resistance to anticancer drugs is afterward evaluated [71]. This model offers the possibility of identifying genetic and epigenetic alterations in PLCs that may underlie the development of drug resistance, thus allowing a link to be established between the genomic profile and chemosensitivity [72,73]. This approach can predict the potential clinical benefits of anticancer agents for each tumor [74,75], and hence improve the design of efficient and personalized therapies against PLCs and their recurrences. Nevertheless, primary cultures of cancer cells also have some limitations, such as the availability of the specimen, because carrying out a biopsy is not always recommended, the small size of the available tissue and the technical difficulties to isolate and culture these cells. This model has been used to characterize the sensitivity of various types of CCA to drugs such as gemcitabine and cisplatin that are commonly included in their pharmacological regimens [76].

In a study carried out using primary cultures of HCC cells, the authors identified the differential expression of several genes involved in proliferation, remodeling of the extracellular matrix, migration, implantation, immune escape, or angiogenesis, and hence affecting chemoresistance [72].

A variant of primary culture of cancer cells consists of the selection of CSCs, which are believed to play an essential role in the failure of long-term chemotherapy because of their marked MDR phenotype and the fact that they can remain mitotically inactive in hypovascular regions of solid tumors, due to specific characteristics absent in healthy stem cells, which include hyper-efficient DNA repair mechanisms, overexpression of anti-apoptotic proteins, resistance to a hypoxic microenvironment, and overexpression of ABC pumps [77,78]. In HCC, several populations of CSCs expressing specific biomarkers for prognosis, chemoresistance, and metastasis, have been identified [79]. In CCA, the presence of CSC markers has also been reported [80]. Among them, the expression in intrahepatic CCA of SOX9 [81] and ID3 [82] has been associated with the response to chemotherapy and enhanced chemoresistance, respectively.

Coculture of liver CCLs with other cells as occurs in the in vivo situation has revealed that the tumor environment can both enhance the sensitivity and promote the chemoresistance depending on the cell type, used drug, and cellular environment composition. For example, coculture of human hepatoma HepG2 cells with the human hepatic stellate cell line LX2 leads to inhibition of p53 activation [83], a frequent event in HCC that makes tumor cells refractory to activate apoptosis in response to chemotherapy [84], thus protecting HepG2 cells against cisplatin-induced apoptosis [83]. Besides, hepatic stellate cells coculture confers sorafenib resistance to Huh-7 cells through two independent pathways HGF/c-Met/Akt and Jak2/Stat3 [85].

Remarkable progress in the study of proteins involved in chemoresistance has come from studies carried out using heterologous expression systems—like bacteria, yeasts, insect cells, or *Xenopus laevis* oocytes—which offer the benefits of easiness of genetic manipulation. Thus, the functional expression of human ABC pumps and solute carrier transporters in *Xenopus laevis* oocytes has permitted the identification of several of their drug substrates, such as sorafenib for OCT1 [51], SN-38 for OATP2B1 [86], mitoxantrone for BCRP [87], and 6-mercaptopurine for MRP4 [88]. This model has also enabled analysis the relevance of mutations on the expression and function of MOC-1 proteins, for example, R61S fs*10 and C88A fs*16 variants of OCT1 [51] and Y556C and V776I variants of MRP4 [88] that alter their substrates disposition.

## 4. Spheroids and Organoids

Because in vitro 2D cultures of CCLs fail to accurately recapitulate the characteristic features of liver tumors—such as three-dimensional architecture, cell-to-cell, and cell-to-matrix interactions, cellular heterogeneity, and the effect of the tumor microenvironment—alternative multicellular 3D models, such as spheroids and organoids have been developed. Spheroids are established from CCLs or primary cancer cells cultured under non-adherent conditions [89]. Although spheroids constitute a model closer to the in vivo situation, they do not fully mimic tumor circumstances because there is a lack of cell heterogeneity. Nevertheless, this model has been useful to advance in the knowledge of cancer chemoresistance. Thus, spheroids display a higher expression than CCLs in 2D cultures of CSC markers, which have been associated with chemotherapy resistance and poor outcome in HCC patients [90] and with resistance to cisplatin/gemcitabine in CCA patients [91]. Together with changes in pH or hypoxia, metabolites released by stromal cells may affect the expression of proteins in spheroids and induce chemoresistance. Thus, epithelial–mesenchymal transition related genes were expressed at much higher levels in co-cultures of mouse hepatoma Hepa1-6 spheroids and mouse liver JS-1 stellate cells, and this was accompanied by a higher expression of TFG-β1 and resistance to paclitaxel [92].

On the other hand, tumor organoids—also known as tumoroids or tumorospheres—are promising tools to investigate potential targets for new drugs and to identify prognostic biomarkers and mechanisms involved in inherent and acquired resistance to chemotherapy. Usually, tumor organoids are obtained by mechanical dissociation of tumor tissue [93], which accounts for a higher order in the self-assembly of cell organoids as compared to spheroids. Besides, the dependence on the extracellular matrix is higher for organoids than for spheroids. Although organoids derived from other human tumors have been previously obtained [94,95,96], the generation of organoids from resected HCC, CCA, and combined hepatocholangiocarcinomas [97] and ultrasound-guided tumor needle biopsies obtained from HCC and CCA [98] has been only recently reported and the number of publications is still limited. All these tumoroids were derived from tumors poorly differentiated with high cell proliferation rates. For months, they maintained the gene expression patterns and the genomic alterations characteristic of their tumors of origin. Moreover, tumoroids retained the histological characteristics of the original tumors both when they were cultured in vitro and subcutaneously implanted in immunodeficient mice [97], where they displayed marked tumorigenicity. Tumoroids have been used in studies of chemoresistance, to compare the sensitivity to different anticancer drugs, revealing enhanced resistance to most assayed drugs [97]. Moreover, a correlation between the sensitivity to certain drugs and mutational profiles in genes codifying their molecular targets was found. For example, the porcupine inhibitor LGK974 reduced the growth of tumoroids expressing wild-type Wnt/β-catenin, but had no effect on tumoroids with mutations in the *CTNNB1* gene [97].

Using patient-derived organoids that were established from the liver biopsy of an individual with metastatic chemoresistant intrahepatic CCA, the lack of sensitivity to the combined therapy of 5-FU and oxaliplatin that the patient had previously received was confirmed. Moreover, the study of these tumoroids could suggest that miRNA-21 was involved in the resistance to HSP90 inhibitors [99].

Initially, only epithelial-derived PLC cells were used to generate tumoroids but, since the microenvironment plays a crucial role in chemoresistance, the incorporation of stromal cells has helped to obtain structures closer to that of real tumors, with typical desmoplasia. Thus, when the HCC cell lines HCCLM3 and Hep3B were cocultured with non-parenchymal cells (fibroblast and endothelial cells) they expressed more neo-angiogenesis-related markers (VEGFR2, VEGF, HIF-α), tumor-associated inflammatory factors (CXCR4, CXCL12, TNF-α) and epithelial–mesenchymal transition-related proteins (TGFβ, Vimentin, MMP9) compared with homogeneous spheroids formed by only HCC cells [100].

Tumoroids can also be generated from healthy organoids by viral transduction or CRISPR/Cas9-mediated genomic edition. These PLC organoids can be used to study the role of mutations affecting MOC genes in drug sensitivity. Recently, organoids from murine extrahepatic CCA were established by duct-cell-specific gene manipulation consisting of the loss of E-cadherin and type 2-TGFβ receptor and Kras activation and this model was used to study mechanisms of biliary injury-based carcinogenesis [101].

## 5. In Vivo Models

Despite advances achieved using the in vitro models, an in-depth understanding of MOCs in PLCs requires the use of animal models reproducing some of the genetic and pathophysiological hallmarks of these tumors (Table 1).

### 5.1. HCC Models

Most common in vivo models of HCC are based on the endogenous generation of the tumor in rodents by treatment with carcinogens, alteration of their diet, genetic manipulation, or implant in immunodeficient mice of xenograft fragments of HCC tumors.

One advantage of chemically-induced carcinogenesis is its similarity with the injury-fibrosis-malignancy steps occurring in humans. These models are developed by exposure to cancer-inducing compounds that are either carcinogens (genotoxic agents able to directly induce cancer) or co-carcinogens and promoting agents that enhance tumor formation in association with carcinogens [102]. Carbon tetrachloride is a toxin that induces HCC development in mice and rats after 30 weeks [103]. Although this model recapitulates key features of human HCC and is useful to analyze the expression of MOC genes, the rate of tumor formation is lower (30%) than in the diethylnitrosamine (DEN) model, in which HCC is developed in most rats and mice after receiving DEN [104]. A drawback of this model is the long-time (more than 50 weeks) required to generate the tumors. This model has been used to determine the expression of genes involved in intrinsic chemoresistance. The results revealed that HCC nodules overexpressed miR-221, which was involved in sorafenib resistance through inhibition of apoptosis (MOC-5) [105]. The “Solt & Farber“ protocol is a modification of the DEN model in rats, which includes partial hepatectomy after initiation with DEN to speed up carcinogenesis resulting in tumors development after 32 weeks [106]. This model has been used to study the expression of drug transporters (MOC-1) in HCC. For instance, the results of a recent study have indicated that OCT1 involved in sorafenib transport is downregulated in DEN-induced HCC, which correlated with impaired sorafenib uptake by cancer cells [107].

The peroxisome proliferator-activated receptors ligands can activate peroxisomal oxidase and induce the generation of reactive oxygen species, which subsequently promotes HCC development. In mice fed with a diet containing peroxisome proliferators, HCCs appear after 50 weeks [108].

A model close to clinical HCC, which could be useful to study the role of MOC-4-genes in HCC chemoresistance is based on the administration of aflatoxin B1. This hepatotoxin, produced by *Aspergillus* fungi, induces chromosomal aberrations, DNA-strand breaks, adducts generation, micronuclei and uncontrolled DNA synthesis [109], which results in the appearance of HCC in mice and rats after 52 weeks of administration with a success rate of almost 100% [110].

Among diet-induced HCC models, the most popular is the use of a choline-deficient diet in mice and rats, which induces steatohepatitis, fibrosis, cirrhosis, and finally HCC after 50 weeks [111], due to the generation in oval cells of oxidative stress, DNA damage, and genetic mutations. This model has been used to analyze the connection between inflammatory pathways and chemoresistance. Thus, a link between TNFα and resistance to sorafenib has been reported, which probably involves the induction of epithelial–mesenchymal transition (MOC-7) [112].

Genetically engineered mouse models have the advantage of mimicking pathophysiological and molecular features of HCC. The most common examples are the transgenic mice expressing oncogenes, such as *MYC* (c-Myc) or *CTNNB1* (β-catenin), and mice with mutation/deletion of several genes such as *PDGF*, *TGFβ1*, *NEMO*, *TAK1*, *A1AT* (α1-antitrypsin), or *PTEN*. Experimental evidence suggests that tumor suppressor genes also play an important role in HCC response to anticancer drugs. For example, PTEN has been related to trastuzumab, cetuximab, gefitinib, and erlotinib resistance by activation of the PI3K pathway (MOC-5) and MRP1 upregulation (MOC-1b) [113]. Besides, sorafenib resistance in HCC has been linked with the inhibition of autophagy via the PTEN/Akt pathway [114]. Accordingly, the liver-specific PTEN-deficient mice model has been used to study chemoresistance associated with the loss of this gene. Genetically engineered mouse models also have some limitations related to the presence of the mutations during embryogenesis, activation of compensatory molecular pathways, and the expression of the transgene in all hepatocytes. Moreover, these models frequently require complex and time-consuming strategies for breeding.

Regarding the use of HCC xenograft models, their main advantages are the rapid induction and easy surveillance of tumor growth. Several CCLs have been used to form subcutaneous xenografts to study chemoresistance. For example, the role of OCT1 in sorafenib uptake was demonstrated in mice using subcutaneously implanted tumors formed by HepG2 cells with and without overexpression of the carrier [107]. Nevertheless, it should be mention that, although HepG2 cells are widely used in HCC models due to their phenotype, and they are sold by the American Type Culture Collection as HCC cells, they were initially obtained from a 15 years-old boy diagnosed from HB.

Orthotopic models have been used to investigate MOCs and evaluate new strategies for HCC treatment. For instance, human Hep3B HCC cells, highly responsive to sorafenib, have been used with this purpose in three different xenograft models, namely, orthotopic and subcutaneous transplant in severe combined immunodeficient mice, and orthotopic implant in athymic mice. The results revealed that the development of resistance to sorafenib was reversible. Moreover, metronomic chemotherapy based on tegafur (an oral 5-FU prodrug) plus sorafenib increased the response by delaying the appearance of such resistance [115]. The orthotopic implant of HCC, previously generated subcutaneously in a donor mouse, has been used as an experimental model to evaluate strategies aimed at overcoming HCC chemoresistance by using targeted agents encapsulated into liposomes [116]. A variant that mimics human HCC consists of the orthotopic implant of HCC cells in fibrotic livers, which induces faster tumor development, and increases the capacity to metastasizing and forming satellite nodules [117]. In the hollow fiber assay CCLs are inoculated into hollow polyvinylidene fluoride fibers that, after being cultured in vitro for 24–48 h, are subcutaneously or intraperitoneally implanted in athymic mice [118]. The main disadvantage of xenograft models is that the pathophysiological processes associated with HCC development do not resemble the main changes occurring in the human liver.

### 5.2. CCA Models

CCA can be induced by treatment with carcinogens, such as dimethylnitrosamine, thioacetamide (TAA), or furan. Other carcinogenic protocols include infection by liver flukes, such as *Opisthorchis viverrini* and *Clonorchis sinensis*. DEN, in combination with phenobarbital added to the drinking water, results in a ductular reaction that progresses to biliary fibrosis and eventually (62% of animals) to HCC and CCA [119]. TAA is usually administered in drinking water to obtaining 100% of success after 12 months of treatment [120]. The TAA model is a multi-step model of carcinogenesis that recapitulates human CCA generation, which is useful to analyze stage-associated intrinsic resistance. The upregulation of the stem cell factor and its receptor c-Kit results in the activation of a proliferative and anti-apoptotic signaling pathway, which has been reported in animals with TAA-induced CCA [121], therefore constituting a useful model to study MOC-5. Because this model is accompanied by intense stromal desmoplasia, it can be used to evaluate the role of the stroma and cancer-associated fibroblasts in CCA chemoresistance (MOC-6). Thus, TAA model has permitted to demonstrate that paclitaxel and nab-paclitaxel (nanoparticle albumin-bound paclitaxel) induced similar anti-proliferative effects via induction of apoptosis in CCA cells *in vitro*, however *in vivo*, using the TAA model, only nab-paclitaxel induced tumor growth inhibition due to its ability to affect surrounding cancer-associated fibroblasts [122].

Because bile acids promote CCA [123], cholestasis alone or in combination with carcinogens has been the basis for several CCA animal models. For instance, partial cholestasis induced by the left and central bile duct ligation and the administration of dimethylnitrosamine or DEN showed early-onset and higher rates of tumor development compared with these carcinogens alone. The accumulation of bile acids in addition to inducing apoptosis also stimulates ERK1/2, Akt, and NF-kB pathways, which enhance cell proliferation, migration, and survival [124].

Genetically engineered mouse models of CCA include those based on frequent oncogenic alterations observed in humans, such as *TP53*, *PTEN*, and *SMAD4* loss, and activation of KRAS, IDH, and NOTCH signaling. One of the most popular models is based on liver-specific deletion of tumor suppressor genes *KRAS* and *TP53*, which recapitulates the multi-stage histopathologic progression of human CCA [125]. In the case of p53, their role in chemoresistance is not entirely known and seems to depend on both cellular context and the type of the administered drug [126,127], while mutated KRAS is a poor biomarker for prognosis in CCA patients [128].

Several studies have used subcutaneous CCA xenograft to investigate the role of transporters in chemoresistance (MOC-1). The results have permitted to propose a temporal drug administration pattern based on the findings that a drug can either increase or diminish the uptake of a second drug due to direct alterations in the transportome [129]. A useful variant to study chemoresistance and test new strategies to overcome this problem has been to generate xenografts using resistant CCA cells, such as QBC939. This approach has enabled evaluation of the role of retinoic acid receptor-β (RARβ) in 5-FU resistance [130], and the potential role of capsaicin to overcome drug resistance to 5-FU [131].

A common problem in the generation of CCA xenografts is the poor tumorigenesis of CCLs, as is the case of SSP-25 cells. To solve this problem, these CCA cells have been implanted using the hollow fiber approach to study the ability of lovastatin and gefitinib co-administration to treat gefitinib-resistant CCA cells [132].

Small fragments of subcutaneously grown tumors in a donor mouse have been implanted in the livers of host immunodeficient mice, for instance, to evaluate a gene therapy strategy to overcome sorafenib resistance [133]. In other studies, CCA cells have been directly seeded into the mouse liver [134] or intravenous delivered through the portal [135] or the splenic [136] veins. In these methods, an abundant stroma is generated, which is enriched in activated fibroblast developed around CCA-derived tumors. These mimic the appropriate conditions to evaluate MOC-6 in CCA. Orthotopic models of CCA present the difficulty of the anatomical characteristics of these tumors, which makes it particularly challenging to simulate extrahepatic CCA. Some authors have inoculated CCA cells (BDEneu) into the bile duct of isogenic rats resulting in tumor growth within the liver, bile duct obstruction, and gross peritoneal metastases [137]. This model recapitulates the extensive desmoplastic reaction typical of human CCA containing cancer-associated fibroblasts.

### 5.3. HB Models

To induce endogenous HB is difficult due to the nature of cells originating this tumor and the time when carcinogenesis is initiated, usually during intrauterine life. Ectopic subcutaneous models of HB have been used to study therapeutic options and chemoresistance. For instance, in the case of pediatric liver cancer patient-derived xenografts, cell suspensions obtained from tumors removed from children suffering from HB were subcutaneously injected into nude mice. The cytogenetic array, histology, and mutational analysis of the parental tumors and the corresponding patient-derived xenografts confirmed high preservation of molecular features seen in the parental tumors [138]. This model has been used to evaluate the role of MDR1 in drug resistance (MOC-1b). With this aim, the animals were treated with or without PSC 833 (an MDR1 inhibitor). The results indicated that this drug significantly improved the effects of chemotherapy with cisplatin [139] and doxorubicin [140]. The orthotopic model of HB in mice, generated with chemoresistant HB cells (HepT1 and HuH-6), has been used to analyze the impact of apoptosis modulation (MOC-5) on drug sensitization [141].

## 6. Conclusion and Future Perspectives

Despite the advantages in the development of experimental models used in the study of resistome, their limitations usually make necessary the combination of several tools to achieve an accurate response to the question under investigation. For instance, although CCLs are excellent models for the study of chemoresistance, their resistome often differs from that of freshly collected tumor cells [142,143,144]. This constitutes an important drawback because the results obtained in CCLs may not accurately replicate MOCs acting in the clinical situation. Accordingly, many compounds that gave promising results when were assayed in vitro failed when they were administered in clinical trials, which highlights the need for other models for understanding MOCs in PLCs. Technical advances permit the use of patient-derived xenograft instead of CCLs to identify the individual resistome of each tumor at each time of its evolution or treatment and hence predict their response to adjuvant chemotherapy and possibly select patients for most effective chemotherapy in each case. With this aim, it would be particularly interesting to identify the resistome signature of CSCs but, unfortunately, most markers described in PLCs are shared by both CSCs and healthy stem cells, thus hampering the specific characterization and the use of targeted strategies for CSCs in these tumors. Another difficulty in the use of in vitro models is their dissimilarity with the real tumor structure, microenvironment, and cellular heterogeneity. The use of cocultures and multicellular 3D approaches—such as spheroids or organoids—can more accurately mimic tumor tissue, as they preserve many of their biochemical and morphological characteristics. This is a fundamental advantage since these factors have a significant influence on gene expression and, therefore, on cellular behavior and chemoresistance. In the framework of personalized medicine and the use of advances in our knowledge of PLC chemoresistance, the combination of subcellular approaches, such as the identification of MDR proteins present in circulating extracellular vesicles and the development of in vivo chemosensitivity assays using organoids, constitute promising tools to translate laboratory results into clinical practice in the near future.

## Figures and Tables

**Table 1:** Synopsis of the key advantages and limitations of the main animal models used in HCC, CCA, and HB and their usefulness in chemoresistance evaluation.

Model	Advantages	Limitations	Usefulness in Chemoresistance Research
Carcinogen-induced tumors	Similarity with the carcinogenesis phases seen in humans	Time consuming high number of animals needed	Intrinsic resistance study of changes in gene expression during carcinogenesis
Genetically engineered mouse models	Facilitate detailed investigation of carcinogenic pathways	Complex and time-consuming breeding strategies	Study of the role of oncogenes and tumor suppressor genes in MDR
Ectopic implants	Fast tumor development Easy to perform No post-operative complications	Important differences between cell lines No interaction with liver tissue Model of advanced tumor stage Absence of metastasis	Drug screening in resistant cells Evaluation of the role of MOC genes using modified cell lines
Orthotopic implants	Fast tumor development allows complex tumor-host interactions to be tested Feasibility to use fibrotic liver	Difficult procedure Important differences between cell lines The need of immunodeficient animals makes less realistic the tumor microenvironment	Drug screening in resistant cells Tumor-host interaction Evaluation of the role of MOC genes using modified cell lines

## Abbreviations

5-FU, 5-fluorouracil; ABC, ATP-binding cassette; BCRP, Breast cancer resistance protein; CCA, cholangiocarcinoma; CCL, Cancer cell line; CSC, Cancer stem cell; DEN, Diethylnitrosamine; HB, hepatoblastoma; HCC, hepatocellular carcinoma; MDR1/P-gp, Multidrug resistance protein 1/P-glycoprotein; MOC, mechanism of chemoresistance; MRP, Multidrug-resistance associated protein; PLC, primary liver cancer; PMVs, plasma membrane vesicles; TAA, Thioacetamide.
